# Development of Highly Flexible Piezoelectric PVDF-TRFE/Reduced Graphene Oxide Doped Electrospun Nano-Fibers for Self-Powered Pressure Sensor

**DOI:** 10.3390/polym16131781

**Published:** 2024-06-24

**Authors:** Arsalan Ahmed, Nazakat Ali Khoso, Muhammad Fahad Arain, Imran Ahmad Khan, Kashif Javed, Asfandyar Khan, Sanam Irum Memon, Qinguo Fan, Jianzhong Shao

**Affiliations:** 1 Department of Textiles and Clothing, School of Engineering and Technology, National Textile University Karachi Campus, Karachi 74900, Pakistan; muhammadfahad@ntu.edu.pk; 2 Engineering Research Centre for Eco-Dyeing and Finishing of Textiles, Ministry of Education, Zhejiang Sci-Tech University, Hangzhou 310018, China; jshao@zstu.edu.cn; 3 Key Laboratory of Advanced Textile Materials & Manufacturing Technology, Ministry of Education, Zhejiang Sci-Tech University, Hangzhou 310018, China; nazakat.ali@buitms.edu.pk; 4 College of Materials & Textiles, Zhejiang Sci-Tech University, Hangzhou 310018, China; 5 Department of Textile Engineering, Balochistan University of Information Technology, Engineering and Management Sciences (BUITEMS), Quetta 54000, Pakistan; 6 Department of Textile and Apparel Science, School of Design and Textile, University of Management & Technology, Lahore 42000, Pakistan; imran.ahmad@umt.edu.pk (I.A.K.); kashif.javed@umt.edu.pk (K.J.); 7 Department of Textile Engineering, Daffodil International University, Dhaka 1216, Bangladesh; 8 Textile Engineering Department, Mehran University of Engineering & Technology (MUET), Jamshoro 76062, Pakistan; sanam.irum@faculty.muet.edu.pk; 9 Department of Bioengineering, University of Massachusetts Dartmouth, North Dartmouth, MA 02747, USA; qfan@umassd.edu

**Keywords:** biomedical, conductive polymer, dielectric response, piezoelectric, pressure sensor, self-powered material

## Abstract

The demand for self-powered, flexible, and wearable electronic devices has been increasing in recent years for physiological and biomedical applications in real-time detection due to their higher flexibility and stretchability. This work fabricated a highly sensitive, self-powered wearable microdevice with Poly-Vinylidene Fluoride-Tetra Fluoroethylene (PVDF-TrFE) nano-fibers using an electrospinning technique. The dielectric response of the polymer was improved by incorporating the reduced-graphene-oxide (rGO) multi-walled carbon nano-tubes (MWCNTs) through doping. The dielectric behavior and piezoelectric effect were improved through the stretching and orientation of polymeric chains. The outermost layer was attained by chemical vapor deposition (CVD) of conductive polymer poly (3,4-ethylenedioxythiophene) to enhance the electrical conductivity and sensitivity. The hetero-structured nano-composite comprises PVDF-TrFE doped with rGO-MWCNTs over poly (3,4-ethylenedioxythiophene) (PEDOT), forming continuous self-assembly. The piezoelectric pressure sensor is capable of detecting human physiological vital signs. The pressure sensor exhibits a high-pressure sensitivity of 19.09 kPa^−1^, over a sensing range of 1.0 Pa to 25 kPa, and excellent cycling stability of 10,000 cycles. The study reveals that the piezoelectric pressure sensor has superior sensing performance and is capable of monitoring human vital signs, including heartbeat and wrist pulse, masticatory movement, voice recognition, and eye blinking signals. The research work demonstrates that the device could potentially eliminate metallic sensors and be used for early disease diagnosis in biomedical and personal healthcare applications.

## 1. Introduction

Physical body signals such as conscious and unconscious muscle movement, including changes in muscle status, reflect early signs of potential human health issues [1]. In the ICU (intensive care unit), especially for patients who have gone through a tracheotomy, weak swallowing strengths may cause saliva to go to the lungs, thus inducing pneumonia [2]. For instance, coughing is the most common symptom for which individuals seek medical advice [3]. For spine trauma patients, the strength of breathing or coughing may indicate a recovery state. Such types of human body signals need to be monitored closely, preferably autonomously [4]. Besides acute and clinical patients, the daily health status of outpatients should also be monitored wirelessly so that the number of follow-up visits to doctors can be reduced and thus save healthcare resources [5]. For instance, the number of patients recovering from anesthesia is incredibly large, but their recovery requires careful monitoring [6]. To fulfill the demand for acquiring massive health data from body signals, this research will explore feasible methods for data collection as well as analysis to deliver relevant critical health information using energy harvesting systems as energy-efficient, intelligent, and autonomous sensing systems. Energy harvesters convert ambient energy in the working environment, including optical, kinetical, and thermal energy, into electrical signals [7]. Therefore, energy harvesters are naturally smart sensors that can dynamically respond to ambient stimuli. In this research, the stimuli will be representative muscle movements triggered by coughing, breathing, swallowing, etc. The methodology of this research is to collaborate with clinics and/or medical researchers to figure out a series of appropriate body signals that can be reliably detected by energy harvesting systems and thus can be potentially used as health data, which helps to predict and diagnose early diseases and health status, respectively [8].

Flexible and wearable artificial electronic skin (e-skin) as a pressure sensor is capable of converting human skin sensory functions from mechanical stimuli, for example, pressure, movement, and body temperature, into electrical signals [9]. The human skin-inspired pressure-sensitive e-skin has received significant attention in recent years as a highly sensitive device for biomedical and health monitoring [10]. The artificial e-skin must have the capability to detect the variable pressure during physiological movement and deformation in the form of dynamic and static stimuli with higher accuracy [11,12]. Moreover, it is essential to obtain detection parameters such as pulse rate, heartbeat, and muscle movement from various parts of the human body through e-skin for real-time health monitoring, fulfilling daily life requirements. For several decades, the development of organic and inorganic materials has been used for the design and development of wearable smart devices [13]. The organic materials include carbon, carbon nano-tubes, graphene, and its derivatives [14]. Whereas inorganic materials, for example, Pb(Zr, Ti)O_3_ (PZT), ZnO, and BaTiO_3_, gold, silver, and copper, have been studied and extensively used for highly sensitive pressure sensors [15,16]. Conductive polymers, in combination with both organic and inorganic materials, are being employed in the advancement of sensitive electronic devices, embedded wearable electronic skins, textiles, and smart gadgets [17]. These wearables are based on self-centeredness, and their sensing mechanisms are also based on capacitive [18], resistive [19], piezoelectric [20], and triboelectric [21]. However, the ultra-sensitivity of the pressure ranges from low-pressure to medium-pressure regime [(<10 KPa) to (10–100 KPa)] respectively. Therefore, there is a high demand for self-powering devices with high flexibility and durability, cost-effective fabrication, and potential end-use [11,12,13,14]. However, the utilization of inorganic materials in wearable e-textiles is constrained due to their inherent characteristics such as high brittleness, density, and limited flexibility, making them less capable of withstanding external forces and bending.

Polymers like polyamides [22], nylon-11 [23], polyurea and liquid crystal [24], polylactic acid (PLA) [25], poly (lactic-co-glycolic acid) (PLGA) [26], are commonly used in wearable textiles. Organic carbon fibers and carbon-coated textiles are gaining attention over metallic wires due to their potential to enhance piezoelectric properties, higher flexibility, and ease of preparation. The mechanical strength, chemical stability, biocompatibility, and impact resistance of organic materials make them promising for future sensory applications [24]. These attributes of dielectric piezoelectric materials make various polymeric materials suitable candidates for the fabrication of piezoelectric pressure sensors. However, the piezoelectric effect may produce a poor electrical signal under a low mechanical force and is attributed to the piezoelectric properties of the polymer [27]. Among dielectric materials, the poly(vinylidene fluoride) (PVDF) polymer achieved great importance and compatibility. Tetra Floroethylene (TrFE) has been considered a prompt piezoelectric co-polymer material in the past decade due to its unique and outstanding piezoelectric properties attributed to the β-crystallization phase [27]. In the PVDF-TrFE chain, if the dipoles in the β-piezoelectric crystallization phase are entirely oriented in the same direction, the largest piezoelectric outputs are produced by the piezoelectric polymer [28]. However, it is very hard to entirely transform the non-polar α-phase into the β-polar crystalline phase [29]. The transformation of the β-piezoelectric phase is achieved through various techniques, wherein the molecular chains are reorganized into the random arrangements of the trans structure. This effect may be attained to improve the piezoelectric effect through alteration of the β-phase through the incorporation of new material, the orientation of molecular chains by mechanical means or thermal annealing [30,31], surface charge [32,33], uni-axial or bi-axial mechanical stretching effects [34,35,36,37,38], rolling [39], high-pressure quenching of molten [40], under high electrical field [38,41,42], electrospinning [43,44], and doping of nano-fillers [45,46,47,48,49,50]. The electrospinning method has fascinated interest in producing nano-fiber mats [51,52,53]. The β-crystal phase in PVDF-TrFE occurs due to the high stretching polymer ratio of the jets and a high voltage applied to the precursor during the electrospinning process [24,54]. The viscosity of the solution also greatly influences the properties of the resultant fibers during electrospinning. Wherein the higher aspect ratio of stretching is required for the uni-axial mechanical stretching, which leads to the transformation of α to β-phase [55]. The quick evaporation of solvents such as acetone and the low temperature over a range of 21–59 °C. Whereas, at low temperatures, crystallization and stretching of polymeric chains occur during the spinning of jets and result in an improved transformation of the β-crystal phase of the polymer [56]. Additionally, nano-fillers have been reported in previous studies to show an increase in the β phase for dielectric behavior and signal response with enhanced mechanical properties of the electrospun nano-fibers.

Among the wide variety of organic materials investigated, e.g., silver, copper, gold, nanoclay [57,58,59], organo-silicate composite [60] and inorganic materials, for example, carbon-based nano-materials such as two-dimensional (2D) graphene nano-sheets [61], rGO, and one-dimensional (1D) carbon nano-tubes and nano-wires [62,63] have been reported to enhance the β-piezoelectric phase and orient the polymer assembly, consequently enhancing the piezo-electric effect of the polymer [64,65,66,67]. However, there has been limited research on the doping method of carbon-based fillers during the electrospinning process. These organic fillers are well-suited due to their exceptional physical properties, such as high electron mobility, extensive surface area, improved compatibility, and mechanical properties. The demand for the potential use of PVDF and its copolymer is increasing for various industrial applications, especially piezoelectric pressure sensors [63].

The current study presents an economical and large-scale method for creating a highly flexible and sensitive multi-mode pressure sensor. This sensor incorporates a PVDF-TrFE polymer doped with nano-fibers of reduced graphene oxide (rGO) and multi-walled carbon nano-tubes (MWCNTs) through electrospinning, resulting in a high β-crystal polar phase. Subsequently, the synthesized nano-fibers are coated with an ultra-thin conductive layer of poly(3,4-ethylenedioxythiophene) (PEDOT) via vapor phase polymerization (VPP). Our development involves creating a piezoelectric pressure sensor resembling a skin, utilizing a hybrid nano-composite of rGO-MWCNTs-doped PVDF-TrFE over PEDOT nano-fibers. This sensor exhibits the capability to detect multiple stresses with variable loads and minimal pressure. Its sensing capabilities enable the monitoring of various human physiological activities, including heartbeat, wrist pulse, muscular movement, voice recognition, and eye blinking signals. Furthermore, the developed device’s capability is enhanced for detecting both static and dynamic sensory responses under dynamic loading and unloading conditions.

## 2. Materials and Methods

### 2.1. Materials

All the analytical-grade materials were used as received without further treatment. The polymer (PVDF-TrFE), with a molecular weight of (Mw = 200,000 g mol^−1^), graphite powder (purity 99.95%), and monomer 3,4-ethylenedioxythiophene (EDOT, 99%), received analytical grade from Sigma Aladdin Industrial Inc. Corporation (Shanghai, China). Multi-walled carbon nano-tubes (MWCNTs, purity > 95%) with a diameter of <8 nm were acquired from XFNANO, Inc. (Nanjing, China). Acetone (Ac, 99%) and N,N-dimethylformamide (DMF, 99%) were bought from Sinopharm Chemical Reagent Co., Ltd. (Shanghai, China). All other chemicals were purchased from Sigma Aldrich (Burlington, MA, USA). Iron (III) Chloride (FeCl_3_·H_2_O) as an oxidant was obtained from Beijing Nuoqiyasheng Biotech Co., Ltd. (Beijing, China). The electrospinning set-up was obtained from Beijing Ucalery Technology Development Co., Ltd. (Beijing, China).

### 2.2. Methods

#### 2.2.1. Preparation of Solution

A polymer solution of PVDF-TrFE with a concentration of 21.0% *w*/*w* was prepared by dissolving it in a mixture of DMF and acetone (50:50) at 45 °C. The solution was continuously stirred using a magnetic stirrer for 4 h. The MWCNTs (1.32 g) were mixed with acetone and CNT dispersing agent (TNWIDS, Chengdu Organic Chemical Co., Ltd., Chengdu, China) at a ratio of 8:4:1 by *v*/*v*. The precursor solution was pre-dispersed in rGO/DMF and MWCNTs in acetone; the dispersion was ultrasonically treated for 60 min before adding PVDF-TrFE solution. The rGO-MWCNTs-doped PVDF-TrFE hybrid solution was subjected to ultrasonication for one hour at room temperature to achieve a homogeneous solution. Then the rGO-MWCNTs-doped PVDF-TrFE (RMDP) solution was magnetically stirred at 45 °C for 16 h before electrospinning.

#### 2.2.2. Fabrication of Device

**Figure 1:**
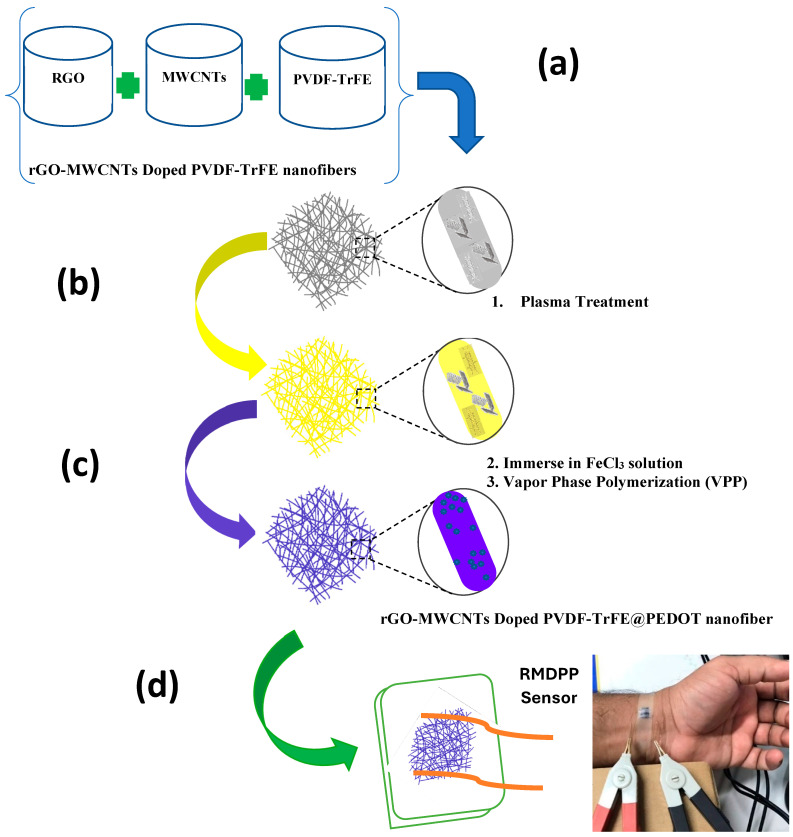
Schematic illustrations for fabrication of nano-fibers: (**a**) rGO, MWCNT, and PVDF-TrFE Solution; (**b**) Plasma treatment and coating of PEDOT via VPP; (**c**) rGO-MWCNTs-doped PVDF-TrFE@PEDOT nano-fiber; (**d**) Fabricated Pressure Sensor.

A schematic of the experiment is presented in Figure 1. The experiment was conducted under optimized conditions on a laboratory scale, with a relative humidity (RH%) of 45% and a closed cabinet temperature maintained at 40 ± 1 °C. The PVDF-TrFE doped with rGO-MWCNTs solution was prepared, loaded in a 10 mL syringe, and mounted with a blunt steel needle with a diameter of 0.028 inches attached to a syringe pump for electrospinning over a high-voltage power source. The nano-fibers were injected from the sharp tip of the electrospinning needle and collected on a horizontally rotating drum (collector) with a constant speed of 130 rpm and an injecting rate of 0.5 mL/h over an applied voltage of 18.0 KV. The collection of nano-fibers was made on the collector, and the distance between the rotating drum collectors and the metal surface electrode was adjusted over a fixed distance of 24 cm from the tip of the needle. Electric high voltage was applied to the capillary syringe and rotating drum with each connector to provide sufficient supply to eject the polymer solution into electrospinning. The resultant fibers were thermally tempered at 100 °C for 2 h to improve the crystallinity of the nano-fiber’s web.

#### 2.2.3. Vapor Phase Polymerization

The obtained RMDP nano-fibers were washed with an ethyl alcohol solution, dried, and then treated with a low-temperature oxygen (O_2_) plasma (input power 200 W) for 80 s and placed in a standard atmosphere for 6 min to induce the formation of reactive functional groups on the fiber surface. Afterwards, the modified nano-fibers were immersed in FeCl_3_.H_2_O and an ethyl alcohol solution (0.276 mol/L) for 15 min and dried at 60 °C for 10 min. Then 200 μL of conductive monomer was placed in conical flasks (three necks). The monomer was preheated to 70 °C to assist the monomer in chemical vaporization. Finally, the monomer was polymerized into conductive polymer (PEDOT) loaded on the surface of the nano-fibers through the Vapor Phase Polymerization (VPP) method under nitrogen gas conditions at a flow rate of 60 mL/s. The nano-fiber-developed sheet was hung over a hook by simple clips and suspended in the chamber for 3 h. After that, the PEDOT-coated nano-fiber sample was taken out of the chamber to eliminate the extra amount of oxidant. At last, the as-coated PEDOT nano-fibers were cleaned several times with Ethanol (EtOH) and simply air dried to obtain conductive nano-fiber sheets.

#### 2.2.4. Fabrication of Piezoelectric Pressure Sensor

The fibrous assembly of the developed nano-fibers was used to fabricate the sensor device, in which both sides of the electrode were connected with adhesive tapes connected with two copper wires at a distance of 0.8 cm from each other, as presented in Figure 1. Finally, the piezoelectric pressure sensor was fabricated with a 1.0 cm × 1.4 cm × 0.03 cm (width and length) for further experimental and performance analysis of the designed sensor. Both ends of the nano-fiber mat were connected with thin copper wires. Then the top and bottom of the hybrid nano-composite mat were covered with PDMS film to ensure the pressure sensor’s isolation from interference from the external environment’s impact on its performance. The PDMS film measures 0.18 μm in thickness. Subsequently, a thin layer of PDMS (Dow Corning Sylgard184; mixed at a ratio of base to cross-linker: 10:1 by mass) was prepared, degassed, and applied to both surfaces of the membranes to encapsulate the device.

## 3. Results

### 3.1. Morphological Analysis (FESEM and TEM)

**Figure 2:**
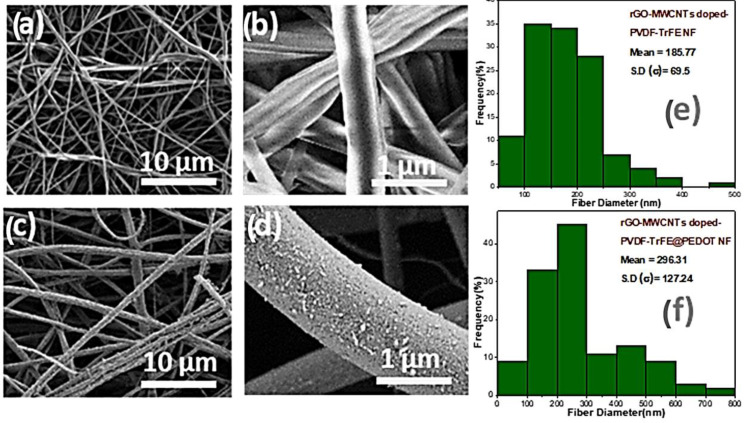
FESEM images of (**a**) rGO-MWCNTs-doped PVDF-TrFE nano-fibers (10 µm) (**b**) rGO-MWCNTs-doped PVDF-TrFE nano-fiber mat (1 µm) (**c**) rGO-MWCNTs-doped PVDF-TrFE@PEDOT nano-fiber mat (**d**) rGO-MWCNTs-doped PVDF-TrFE@PEDOT single nano-fiber (**e**,**f**) The histograms show the diameter distributions of rGO-MWCNTs-doped PVDF-TrFE nano-fibers and rGO-MWCNTs-doped PVDF-TrFE@PEDOT nano-fibers, respectively.

From the Figure 2a,b FESEM image, it was revealed that the fibers were continuous and comprised a well-compact fiber surface morphology. Further, it was also found that there was no bead formation observed in the developed electrospinning condition. The three-dimensional (3D) porous network structure of the doped polymeric nano-fiber mat is well-maintained in Figure 2c. The conductive polymer-coated nano-fibers reveal the presence of meson particles of conductive polymers, as presented in Figure 2d. The FESEM of nano-fiber images revealed a rough surface morphology, which confirms that the coating of conductive polymer nano-particles was attained and is presented in Figure 2d.

The SEM images reveal that the size distribution analysis of rGO-MWCNTs-doped PVDF-TrFE nano-fibers has an average diameter of 185.77 nm (Figure 2e), which is smaller than that of rGO-MWCNTs-doped PVDF-TrFE@PEDOT nano-fibers. The doping of rGO-MWCNTs was performed using electrospinning, followed by VPP of PEDOT on the rGO-MWCNTs-doped PVDF-TrFE nano-fibers, resulting in a noticeable change (Figure 2d). This indicates that the surface of the nano-fibers was coated with PEDOT nano-composites, as confirmed by the increased mean diameter of 296.31 nm (Figure 2f).

**Figure 3:**
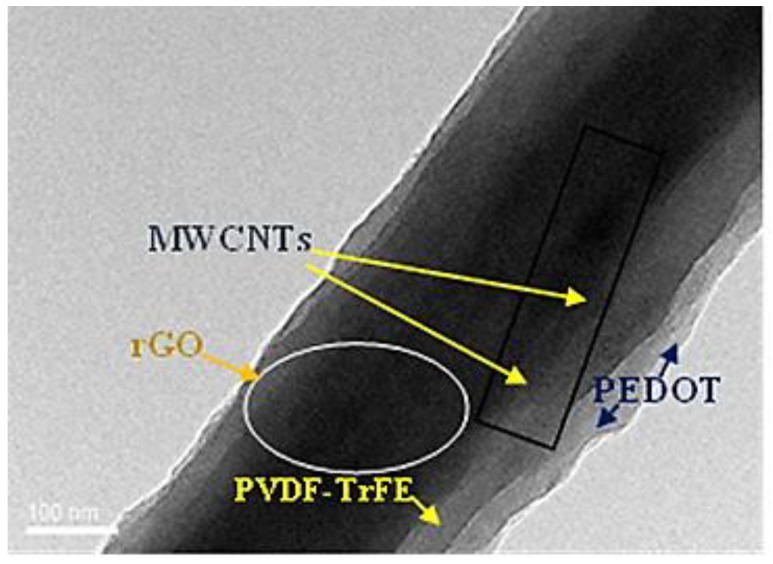
TEM image of rGO-MWCNTs doped with PVDF-TrFE over PEDOT nano-fiber.

From the TEM image, it was found that the noticeable layer of PEDOT on the nano-fiber structure has an average thickness of ~296 nm. Additionally, rGO nano-sheets and multi-wall carbon nanotube MWCNTs inside the nano-fibers were not completely covered by the PVDF-TrFE polymer after electrospinning. However, the fibers were completely wrapped by the conductive polymer as a second layer using vacuum-phase polymerization (VPP), as demonstrated in Figure 3.

### 3.2. XPS Spectra Analysis

**Figure 4:**
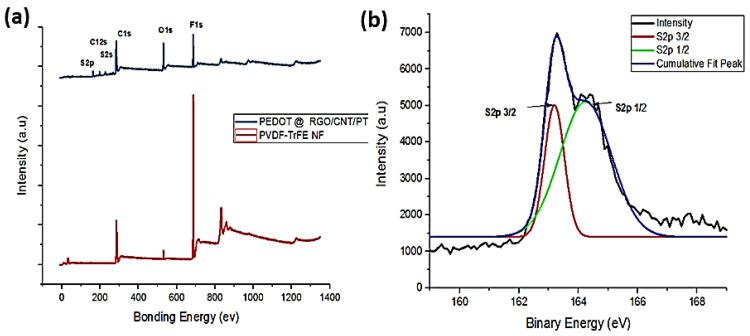
(**a**) XPS survey peaks (**b**) S2P spectrum of rGO-MWCNTs-doped PVDF-TrFE over PEDOT nano-fibers.

XPS spectra were analyzed for the chemical composition of pure electrospun nano-fibers and after conductive polymer-coated nano-fiber mats. Figure 4a shows the typical XPS survey peak of carbon (C), fluoride (F), oxygen (O), and sulphone group (S). It is revealed from the XPS spectra that pristine nano-fibers showing the F-signal peaks reduced after the conductive polymer was coated on as-developed fibers. Whereas the O-signal peaks became prominent and a trace amount of S-signal also appeared. Furthermore, the XPS S2p of the carbon atom shows the de-convolution spectra of the resultant fibers assigned at 163.4 eV and 164.8 eV, which are related to the binding energy levels of the S2p3/2 and S2p1/2 states for the conductive polymer and PVDF-TrFE polymer, respectively, as reported in previous work, Figure 4b [68].

### 3.3. Contact Angle

**Figure 5:**
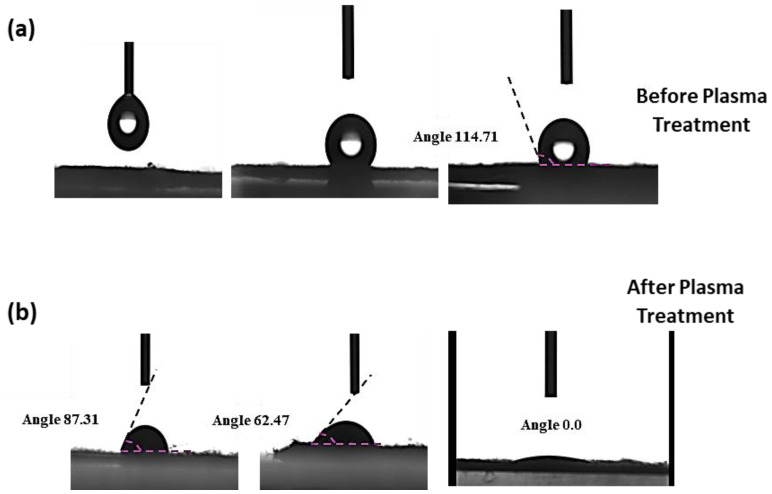
Contact angle of rGO-MWCNTs-doped PVDF-TrFE nano-fibers before (**a**) and after (**b**) plasma treatment.

The developed nano-fibers were prepared and loaded with rGO-MWCNTs via a doping process within PVDF-TrFE nano-fibers. These fibers were subsequently subjected to low-temperature O_2_ plasma to introduce reactive functional sites, such as hydroxyl (OH) and oxygen-containing groups, onto the fiber surface. This synergistic effect could potentially alter the attributes of the nano-fiber mat from hydrophobic to super-hydrophilic. The change in surface modification after plasma treatment was confirmed by testing the water contact angle. The results indicate that the fibers showed a less or negligible water absorption phenomenon with a change in contact angle from 114.7° to 0.0° before and after plasma modification, as reported in previous works and can be depicted in Figure 5a,b. Therefore, the sufficient adsorption of the oxidant FeCl_3_.H_2_O results in speeding up the vapor absorption of the conductive polymer during VPP. However, the bonding that appeared among the PVDF-TrFE nano-fibers and conductive polymer PEDOT was improved by increasing the processing time [69].

### 3.4. Resistivity

**Figure 6:**
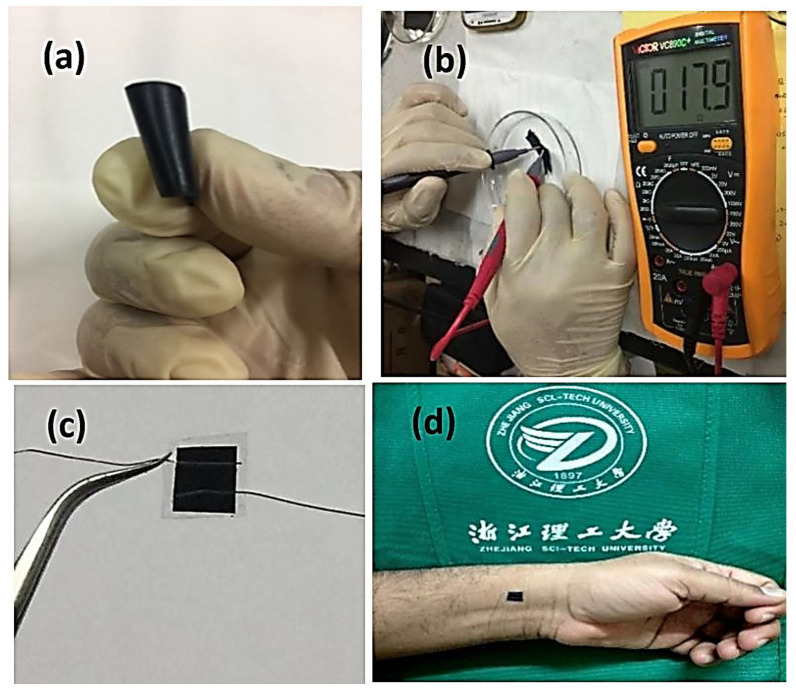
(**a**) Flexible pressure sensor (**b**) resistivity measurement by two-point probe manual ohms meter device and (**c**,**d**) a photograph of a fabricated sensor attached to the wrist of a subject.

The resistivity of the coated and uncoated samples was performed using ASTM Standard (ASTM F43-99) four-point probe techniques as reported in previous studies [70]. In the coated samples after the VPP of PEDOT, the fibrous mat changed its color from white to pale yellow as compared to rGO-MWCNTs-doped nano-fiber mats. However, the color was different and converted to blue-green after the successful deposition of the EDOT monomer and polymerization process established on the substrate nano-fiber mats. The synergistic effect was further verified by the pure PVDF nano-fibers doped with rGO and MWCNT, which showed a lower resistivity of the electrode with a surface resistivity of 2.5–8.5 MΩ/sq. This is further enhanced to 2.5–0.5 Ω/sq by using a 4-point probe resistivity tester according to ASTM F43-99, as demonstrated in Figure 6 [70].

### 3.5. Durability

**Figure 7:**
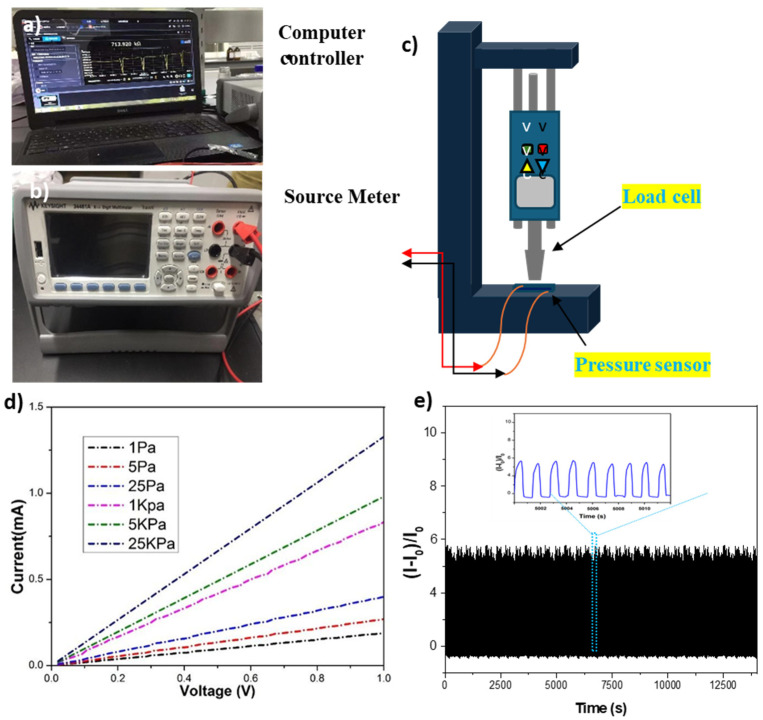
Experimental setup showing (**a**) PC controller (**b**) digital multi-meter (**c**) Mechanical set up for pressure load (**d**), I–V curves of developed pressure sensor under different pressure ranges (**e**) stability measurement record of repeated dynamic and static cycles at a frequency of 20 Hz and a pressure of 25 kPa.

**Figure 8:**
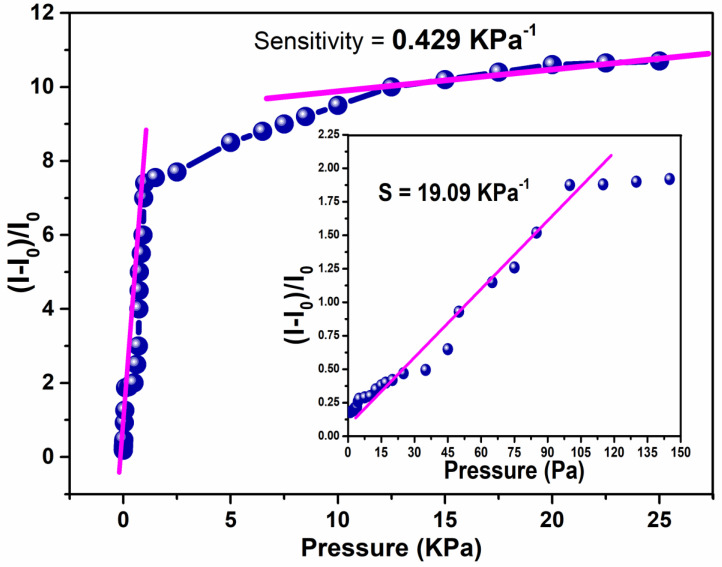
The sensitivity response of the sensor of fibrous mats under applied pressure.

The performance stability and mechanical durability of the developed pressure sensor demonstrate a stable and dynamic response over various frequencies under static and dynamic pressure responses for various force sensing properties, as demonstrated in Figure 7 and Figure 8. The study also shows that the resultant nano-fiber mats with the ultra-fine coating of a conductive PEDOT layer established on the fiber surface may be attributed to higher sensitivity due to the enabling of a 3D structure with more contact points of an individual fiber in the conductive network. This resulted in improved electrical signal response over variable testing and performance under dynamic and static pressure analysis. The effect also leads to a fine and tunable contact sheet resistance towards better capturing of touch response, folding and unfolding, and bending over a wide range under applied minimum pressure. Therefore, it is revealed from the test results performed for various bio-medical signal responses, including voice recognition when placed on the throat, eye blinking, pulse rate, heartbeat, blood pressure, and breathing rate. The developed fibrous micropressure sensor, resulting in high sensitivity and a varied range of multi-modal mechanical sensing capabilities, showed remarkable flexibility for the nano-fiber-based pressure sensor.

### 3.6. The Current–Voltage (I–V)

In the experimental setup linear Figure 7a–c, a motor was utilized to supply precise and periodic compressive pressure that was measured and calibrated by the load cell. The resultant I–V curve of the developed sensor shows good resistive behavior over a variable pressure and gives a highly stable response. The output voltage was significantly increased with increasing the pressure from 1 Pa to 25 kPa. The I–V response was changed from 0.0 V to 1.0 V, as shown in Figure 7d. In brief, during the mechanism, a linear vertical motor tip came into contact with the surface of the pressure sensor. Whereas, when initially the sensor did not show any deformation in the response, the result at the initial current was 0.0 A and an applied voltage of 0.0 V. After that, the vertical motor continuously compresses the sensor down, resulting in the pressure-induced current change (I_0_). Due to this, the distortion of the sensor was enhanced, and in the meantime, the thickness of the compressed sensor decreased as a result of resistance changes in the mat and the contact resistance of the electrode because the neighboring conductive nano-fibers became more compacted. As a result, the induced current was increased accordingly, as demonstrated in Figure 7d. Lastly, the deformation reached a maximum point; meanwhile, the induced current also reached a peak value. This phenomenon demonstrates that, with the applied force to the surface of the sensor, the contact area was increased, resulting in a decrease in sheet resistance, which is attributed to the establishment of a more conductive path and leads to an enhanced output voltage. Moreover, the durability and stability of the pressure sensor were verified through repeated dynamic cycles of 10,000 cycles at a frequency of 20 Hz with a maximum pressure of 25 KPa inserted, as presented in Figure 7e. During the repeated loading/unloading process for cycles, the difference in current persisted at the same level, indicating that the designed sensor had comparable cyclic durability and repeatability.

### 3.7. Sensing Performance

The sensitivity performance was found to be stable for the piezoelectric pressure sensor under changeable vertical mechanical pressure. The relative change in current (I − I_0_/I_0_) was examined by plotting the obtained values as a function with variable applied pressure at 1.0 V. The sensitivity of the piezoelectric pressure sensor and the resultant curve are plotted in Figure 8. The sensitivity performance (S) was calculated using the following formula: S = ((I − I_0_/I_0_)/AP, where ΔI = (I − I_0_) and I_0_ represents a change in induced current in mA with and without loading, and ΔP is equaled to AP as an alteration in the applicable force per unit area (kPa).

The above Figure 8 shows that the sensitivity of the designed sensor showed a broader sensitivity level of 19.09 kPa^−1^ at the initial stage −I with a linear trend of the pressure sensor region, <1 kPa). The results are in agreement and superior to previously reported studies made on pressure sensors [46,47]. Furthermore, it is also expressively superior to the (high-pressure region >1 kPa). In stage II (1–25 kPa), nano-fiber mats were further deformed, and the pressure sensitivity was decreased up to 0.429 kPa^−1^.

### 3.8. Pressure Response (Static and Dynamic)

**Figure 9:**
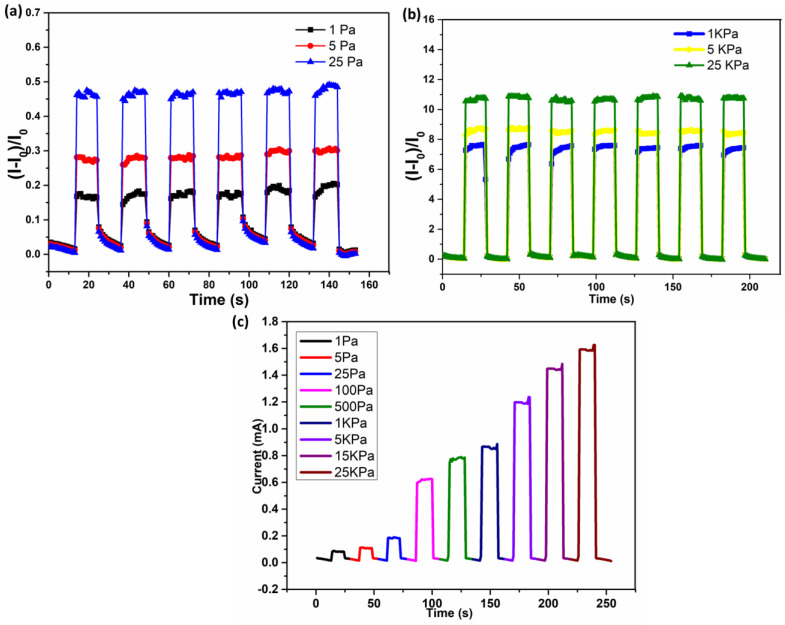
(**a**,**b**) The relative output response of the sensor at very low to high pressure during the first 50–250 cycles, (**c**) the dynamic outcome at varied pressures of 1 Pa to 25 kPa at a constant voltage of 1.0 V.

The sensing properties of the fabricated device were measured at various mechanical stimuli, including pulse, pressure, bending, and speech recognition. The relative changes in current [(I − I_0_)/I_0_] under static and dynamic loading were performed to demonstrate real-time application in human health monitoring. Figure 9a presents the detailed analysis of various responses of sensors from 1 Pa to 25 kPa. The sensor showed variable changes in output current overpressure ranges from 1 Pa to 5 Pa with an applied 1.0 V, as shown in Figure 9b. The results demonstrate that the sensor is capable of very low-pressure detection with higher sensitivity. Furthermore, the sensor exhibited a stable pressure sensitivity over a wide range (1 Pa to 25 KPa) and can be depicted in Figure 9c, which exhibits the dynamic sensor response under variable pressure from 1 Pa to 25 kPa whenever at a constant voltage of 1.0 V, which is subjected to an emergent occurrence at applied pressure, the current was immediately responded to and promptly accumulated to an initial point. Once the applied pressure was separated, the sensor had an estimable and consistent response to sensitivity.

### 3.9. Human Physiological Response and Pulse Rate Response

**Figure 10:**
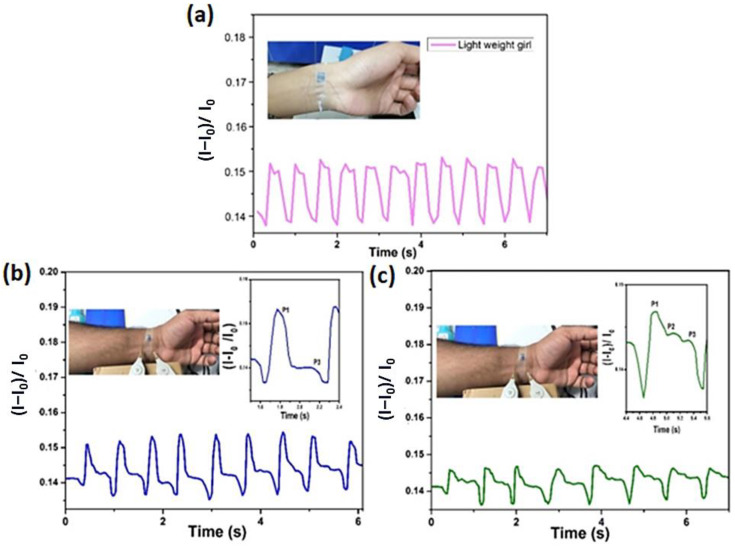
The pulse response signals and images of the sensor placed on the wrist of Subject A, 24 years old, 169 cm, and with 54 kg body weight in the normal state (**a**) and Subject B, (30 years old, 178 cm tall, and 96 kg weight of a male before (**b**) and after (**c**) running condition.

As demonstrated in Figure 10, by nature, the rest of the human body parts and the pulse rate were detected when placed on the human wrist, which indicates arterial pulse generation, which is commonly used for the measurement of blood pressure. The obtained signal response is a highly invaluable subject matter used for the continuous monitoring of human health conditions. In this work, keeping in view the considerations that the sensor is capable of detecting vital signs and could enable to detect or sense very small changes in wrist pulse with high sensitivity, as illustrated in Figure 10a–c. The subtle pressure changes are induced by heartbeats at the wrist pulse. The test results of two healthy subjects, A and B, were 24 years old, 169 cm in height, with a body weight of 54 kg, and 30 years old, 178 cm in height, with a body weight of 96 kg, respectively. Moreover, the prepared piezoelectric sensor has shown high sensitivity to detect very small movements; it could be used magnificently as a wearable E-Skin for prospective practical applications. The sample was attached to human skins employing a polyurethane film dressing (Tegaderm Absorbent, 2.5 M) for detecting subtle stimuli induced by pulse pressure, chewing, facial misexpression, speech recognition, and bending. It is also revealed from Figure 10c that subject B has three peaks (P1–P3), but for subject A there are only two peaks (P1 and P3) (Figure 10a–c). Typically, the wrist artery pulse waveform is found as P1 and P2, which are associated with early and late systolic pressure peaks, respectively, while the diastolic region typically defines P3. The arterial waveform is highly determined by the subject’s age under normal conditions, for example, by different age factors for children, young people, and elder persons, which may also be subjected to gender (Male/Female), healthy and unhealthy (sick) persons, respectively [69]. The heart rates of A and B are 84 bpm and 72 bpm (beats per minute (bpm), respectively. By attaching the sensor to the wrist, the wave signal changes [(I − I_0_)/I_0_] of the output current signal of the sensor. Furthermore, Figure 10a–c shows that the only obvious wrist pulse pressure signal is detected by the sensor, but the two subjects’ amplitude of the subtle pulse was differentiated between the subjects’ body conditions. These results further confirm that the wrist pulse rate frequency of a healthy subject A was 84 beats/min and B was 72 beats/min under normal conditions, respectively.

In addition, we measured the wrist pulse response under different conditions for the same healthy subject, B. After 10 min of running, it was observed that the heartbeat increased from 72 bpm to 126 bpm, the shapes of the wrist pulse wave had changed, and the peak P2, which corresponds to the late systolic shoulder peaks, had diminished, as depicted in Figure 10b. The possible reason is due to the large artery stiffness, which changes the heart rate, and dilated muscular arteries [71].

**Figure 11:**
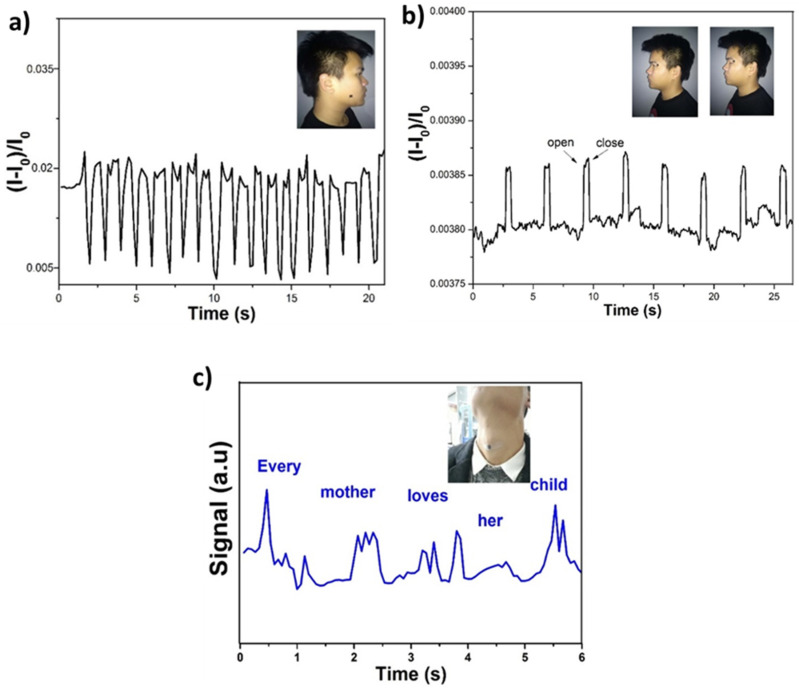
The pressure sensor is used for (**a**) monitoring of the masticatory movement of a human face; (**b**) movement of eye blinking; and (**c**) pressure sensor attached to the human throat subject speaking, “Every mother loves her child”.

Additionally, to monitor the human skin’s subtle motion, the sensor presents high sensitivity. The sensor was attached to the human face to sense the signals of the masticatory movement. Upon repeated subject movements, the pressure sensor promptly generated a current response, as depicted in Figure 11a, particularly during masticatory (chewing) movements. The repeated chewing of the subject caused repetitive changes in an output signal. These results confirmed that the prepared piezoelectric pressure sensor is suitable for monitoring muscular movements and vital signs in human skin. The piezoelectric pressure sensor was used to detect the blinking micro-expression in the eye of the young male subject.

The device was then placed near the eye by detaching with polyurethane film as an adhesive masking tape to monitor the change in blinking expression, as demonstrated in Figure 11b. When an eye was blinked to open and close, the sensor responded instantly, and the relative current change [(I − I_0_)/I_0_] was observed. When observations were made during the blinking of an eye and variable states, i.e., closed and open eye state. Repetitive blinking of a change in the current signal response confirms that the sensor has admirable stability and repeatability. So far, few studies have been previously reported on wearable sensors employed in speech recognition and investigating the delicate muscle movement of the male subject during speech. The device was also attached to the throat to analyze the speaking behavior; as illustrated in Figure 11c, when the volunteer speaks a sentence, for example, “Every mother loves her child”, the pressure sensor exhibited distinct signal patterns, and these subtle pressure changes were induced as a result of complicated muscle movement of the throat. The trails of test performance further demonstrated that different pronunciations correspond to distinctive and repeatable wave signal patterns in each curve for each word. The results confirmed that the fabricated piezoelectric pressure sensor could provide a reliable and effective method for speech recognition and can potentially be used for different kinds of human activities due to its versatility and potential for biomedical applications.

So far, few studies have been previously reported on wearable sensors employed in speech recognition as per data from the Table 1. To capture the subtle muscle movements of a male subject during speech, a pressure sensor was attached above the subject’s throat to measure electrical signals, as shown in Figure 11c. When the subject spoke the sentence ‘Every mother loves her child’, the pressure sensor displayed distinct signal patterns caused by the intricate muscle movements of the throat. We observed that different pronunciations produced unique and repeatable wave signal patterns for each word. These results demonstrate that the fabricated piezoelectric pressure sensor offers an efficient method for speech recognition and can be applied to various human activities due to its versatility and potential for biomedical applications.

## 4. Conclusions

This study involved the preparation of a flexible, self-powered, and wearable piezoelectric pressure sensor composed of a hetero-structured hybrid nano-composite. The pressure sensor was developed by electrospinning PVDF-TrFE doped with rGO and MWCNTs and further coated with the conductive polymer PEDOT through VPP. The exclusive features of the pressure sensor offer 3D conductive paths that provide maximum interaction points and excellent results by capturing a relative change in current induced by pressure. The fabricated nano-fiber-based piezoelectric pressure sensor exhibits ultra-high sensitivity (19.09 kPa^−1^), and high stability over 14,000 cycles with a low operating voltage of 1.0 V and a wide-pressure range from 1 Pa to 25 KPa sensing performance. Additionally, the nano-fiber-based sensor exhibits high sensitivity and is well-suited for real-time health monitoring, including tracking blood pressure and pulse rate. The device is also effective in detecting minor physiological signals such as eye blinking, speech expressions, chewing movements, and vocalizations. The results demonstrate the viscoelastic properties of PVDF-TrFE polymers and their exceptional sensitivity to pressure sensor response, attributed to the dielectric effect induced by the doping of rGO and MWCNT into the polymer. Likewise, conductivity and sensitivity are characteristic outcomes of coating the fibrous mat with a conductive polymer using vapor phase polymerization. We believe that the facile design of the developed piezoelectric sensor is economically viable and highly suitable for multi-mode mechanical sensitivity. It has the potential to be utilized in various wearable microdevices for human health monitoring, biomedical diagnostics, and human–machine interfaces in the future. 

## Figures and Tables

**Table 1:** Summary of the sensor characteristics of different materials.

Material	Form	Sensitivity	Reference
PVDF-TrFE/MWCNTs	Nano-fiber	540 (mV/N)	[72]
PVDF-TrFE@PEDOT	Nonwoven	18.376 kPa^−1^	[73]
PVDF/CNT	Nano-fiber	2.26 (mV/N)	[74]
CNT/rGO doped PDMS	Nonwoven	11.02 kPa^−1^	[69]
PVDF-TrFE	Nano-wire	458.2 (mV/N)	[75]
GO doped PU@PEDOT	Nano-fiber	20.6 KPa^−1^	[18]
rGO-MWCNTs doped PVDF-TrFE@PEDOT	Nano-fiber	19.09 kPa^−1^	Present study

## Data Availability

The original contributions presented in the study are included in the article, further inquiries can be directed to the corresponding authors.
